# Olfactory Dysfunction Predicts 5-Year Mortality in Older Adults

**DOI:** 10.1371/journal.pone.0107541

**Published:** 2014-10-01

**Authors:** Jayant M. Pinto, Kristen E. Wroblewski, David W. Kern, L. Philip Schumm, Martha K. McClintock

**Affiliations:** 1 Section of Otolaryngology-Head and Neck Surgery, Department of Surgery, The University of Chicago, Chicago, Illinois, United States of America; 2 Department of Health Studies, The University of Chicago, Chicago, Illinois, United States of America; 3 Department of Comparative Human Development and The Institute for Mind and Biology, The University of Chicago, Chicago, Illinois, United States of America; 4 The Center on the Demography and Economics of Aging, National Opinion Research Center, The University of Chicago, Chicago, Illinois, United States of America; Technical University of Dresden Medical School, Germany

## Abstract

Prediction of mortality has focused on disease and frailty, although antecedent biomarkers may herald broad physiological decline. Olfaction, an ancestral chemical system, is a strong candidate biomarker because it is linked to diverse physiological processes. We sought to determine if olfactory dysfunction is a harbinger of 5-year mortality in the National Social Life, Health and Aging Project [NSHAP], a nationally representative sample of older U.S. adults. 3,005 community-dwelling adults aged 57–85 were studied in 2005–6 (Wave 1) and their mortality determined in 2010–11 (Wave 2). Olfactory dysfunction, determined objectively at Wave 1, was used to estimate the odds of 5-year, all cause mortality via logistic regression, controlling for demographics and health factors. Mortality for anosmic older adults was four times that of normosmic individuals while hyposmic individuals had intermediate mortality (p<0.001), a “dose-dependent” effect present across the age range. In a comprehensive model that included potential confounding factors, anosmic older adults had over three times the odds of death compared to normosmic individuals (OR, 3.37 [95%CI 2.04, 5.57]), higher than and independent of known leading causes of death, and did not result from the following mechanisms: nutrition, cognitive function, mental health, smoking and alcohol abuse or frailty. Olfactory function is thus one of the strongest predictors of 5-year mortality and may serve as a bellwether for slowed cellular regeneration or as a marker of cumulative toxic environmental exposures. This finding provides clues for pinpointing an underlying mechanism related to a fundamental component of the aging process.

## Introduction

Olfaction is a critical, if underappreciated, component of human physiology. Although potentially less dependent on olfaction than many other mammals [1], humans still rely on this ancestral system which plays an essential role in health and behavior. For example, the olfactory system maintains adequate nutrition through appetite and food preferences [2], [3], enables detection of environmental hazards and pathogens, is associated with memory, emotions and intimate social relationships [4], [5] [6] and is linked neuroanatomically with key parts of the central nervous system [7]–[9]. Finally, normal olfactory function depends on cellular regeneration of the olfactory neuroepithelium, bulb and hippocampus [10]–[12], a capacity impaired by telomere shortening which is a hallmark of aging in many systems [13].

Mortality prediction has typically focused on proximate causes of death such as disease [14] and life-limiting conditions such as frailty [15], [16] and dementia, along with associated biomarkers. Indeed, olfactory dysfunction presages major neurodegenerative diseases including Alzheimer's disease and Parkinson's disease, assessed both clinically and post-mortem, and even mild cognitive impairment [17]–[19]. Given that olfaction has multiple roles and relies on peripheral and central cell regeneration, we hypothesized that olfactory dysfunction could be an early integrative indicator of impending death.

To answer this question, we included olfactory function in the National Social Life, Health, and Aging Project (NSHAP), a nationally representative study of community-dwelling older adults [20]–[25]. NSHAP is the first population-based survey to objectively measure olfaction and generated a rich array of health and social information as well as a five year follow-up that determined mortality [26]. We examined whether olfactory dysfunction predicted mortality after 5 years, utilizing the available health and social information to evaluate potential confounders and test putative mechanisms.

## Methods

### Subjects

NSHAP is the first study of social relationships and health of older adults in a probability sample representative of the United States. In 2005–6 (Wave 1) professional interviewers (National Opinion Research Center [NORC]) conducted in-home interviews with 3,005 community-dwelling older adults (1,454 men and 1,551 women) 57–85 years of age living throughout the US [20], [21]. Five years later, data were collected again (2010–11, Wave 2). To investigate health disparities, race was self-identified using standard NIH classifications. American Indian, Asian, or others formed a single category (“Other”). Further details regarding design, data collection, and baseline characteristics of NSHAP respondents are provided elsewhere [20], [21], [27]. The study was approved by the Institutional Review Boards of The University of Chicago and NORC; all respondents provided written, informed consent.

### Assessment of Olfactory Function in Wave 1

Olfactory function was assessed using a validated odor identification test presented using felt-tipped pens [28] (Burghart Messtechnik, Wedel, Germany). To accommodate the survey's logistical and time constraints, five odorants were selected and presented one at a time. Respondents were asked to identify each by choosing from a set of four picture/word prompts in a forced choice protocol [26]; refusals were coded as incorrect (for test details, see [22], [29]). The target odors were rose, leather, orange, fish, and peppermint. The number of incorrectly identified odors yielded an empiric score (normosmia cutoff based on [30]), which was used to categorize severity of olfactory dysfunction: anosmic  = 4–5 errors, hyposmic  = 2–3 errors, and normosmic  = 0–1 error.

### Determination of Mortality at Wave 2

Mortality status at Wave 2 was confirmed either by speaking with the respondent (alive) or by conducting a proxy interview with a family member or neighbor or examining public records or news sources. Cases were pursued to determine whether respondents were likely alive but not accessible for re-interview. Of the 3,005 Wave 1 respondents, 430 were deceased 5 years later and 2,565 were alive (2,261 reinterviewed, 143 too sick to interview, and 161 determined to be alive but unavailable for interview). There were only 10 cases in which it was unknown whether the respondent was living or not; these were excluded from these analyses. An additional 77 respondents were excluded from the analyses because they had missing data in one of the primary independent variables (odor identification, demographics, and comorbidity index), leaving N = 2,918 for analyses.

### Common Mortality Risk Factors

To adjust for factors known to be associated with mortality, we included a number of covariates in our analyses, many of which have also been found to be associated with olfaction. Age was categorized into groups (57–64 years, 65–74 years, 75–85 years) according to the sampling strategy of the overall study [31]. Socioeconomic status was measured by education (highest degree or certification earned) and net household assets (house, cars, or rental properties/businesses owned; financial assets including savings accounts, stocks, and pensions minus outstanding debt). We present models using only education because both measures yielded similar results. Comorbid diseases were assessed with the Charlson Index modified for NSHAP [32]. In addition, respondents reported whether a doctor had ever told them they had a particular disease. Nutrition measures included self-reported taste (excellent to poor), poor appetite based on “During the last week…I did not feel like eating; my appetite was poor” (“rarely/none of the time” to “most of the time”), and body-mass index (BMI). Inability to perform one or more of seven activities of daily living (ADL) quantified frailty (a biologic syndrome defined by weakness, unintentional weight loss, exhaustion, and lower physical activity, resulting from cumulative declines across multiple physiologic systems, and causing higher morbidity and mortality [15]) [33].

Cognitive function (specifically memory and mental arithmetic) was measured with a modified version of the Short Portable Mental Status Questionnaire (SPMSQ) [34]. Self-rated mental health was measured by a standard 5-point scale (excellent, very good, good, fair, or poor). Health behaviors affecting olfaction were current smoking, based on either salivary cotinine level or self-report, and problem drinking [22], [35].

### Statistical Analysis

NSHAP had a 75.5% survey response rate in Wave 1, excellent for a targeted probability sample, and the non-responders were similar demographically to the responders [31]. Estimates of the prevalence and mean values in the US population were based on weights accounting for differential probabilities of non-response and selection. Design-based standard errors were calculated using the linearization method [36] together with the strata and Primary Sampling Unit indicators provided with the dataset. In Wave 2, NSHAP had a 99.7% determination of mortality. Statistical analyses were conducted with Stata Version 13.0 (Stata Corp LP, College Station, Texas, USA).

We treated the degree of olfactory dysfunction (anosmia, hyposmia, or normosmia) or the number of odors incorrectly identified (0–5) as the independent variable and death as the dependent variable in separate analyses. Multivariate logistic regression was used to model the relationship between olfactory dysfunction and covariates of interest at Wave 1 and death at Wave 2. P-values (all two-sided) and 95% confidence intervals were based on the corresponding Wald statistic. The marginal effects of hyposmia and anosmia on the probability of 5-year mortality were plotted, versus both age alone and the percentiles of a composite risk score computed as a linear combination of several factors affecting the likelihood of death (with coefficients estimated from the logistic model). Sensitivity analyses included: (1) refitting the regression models excluding one identification item at a time (to ensure that a single odor was not unduly affecting the results), (2) an analysis of morbidity and mortality, combining those subjects who were too sick to interview in Wave 2 with those who had died, and (3) an analysis that excluded those reporting a history of head trauma or nasal surgery.

To investigate possible non-linear effects of age, we constructed models which included age^2^. This quadratic term was not statistically significant (data not shown) and did not change the estimate of the olfaction effect on mortality. For parsimony, this term was not included in the models presented here. We also determined whether there were gender differences in associations between olfaction and mortality by: (1) fitting separate models for each gender, and (2) including all gender interaction terms in a gender-pooled model. In neither analysis did the magnitude of the olfaction effect change, nor was there evidence for gender differences. Again, models without the interaction terms are presented here for parsimony.

## Results

### Olfactory Dysfunction, Mortality and Diseases

**Figure 1:**
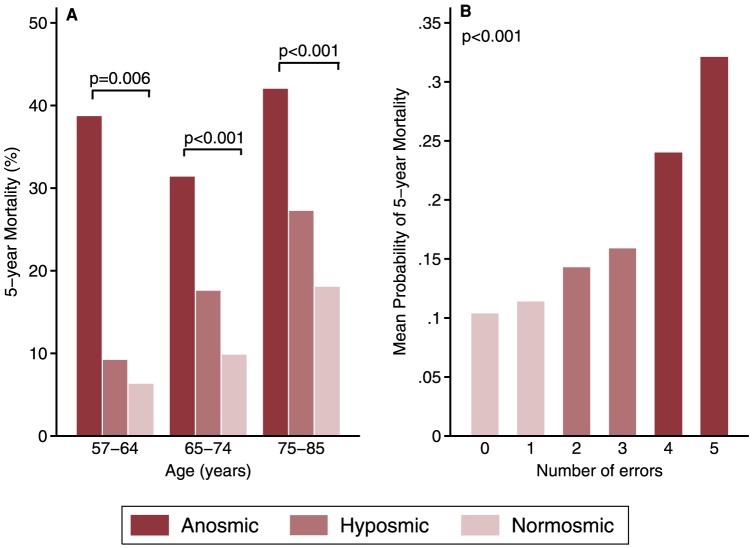
**A.** Olfactory dysfunction and 5-year mortality in three age groups (ages 57–64, 65–74, and 75–85 years). **B.** Progressive increase in 5-year mortality with each additional error in odor identification (p<0.001 from one degree of freedom test for trend); from logistic regression as in Model C Table 2, with number of odor identification errors.

Demographic, olfactory and health characteristics of the US population 57–85 years of age are presented in Table 1. The weighted 5-year mortality rate among the analytic sample was 12.5%, consistent with what would be expected based on the Social Security Administration (SSA) life tables[37]. Fully 39% of older adults with anosmia were dead at Wave 2, 19% of those with hyposmia and only 10% of those with normal olfaction (p<0.001), a dose-dependent pattern seen in all age groups (Figure 1A). This translated into a strikingly increased odds of death for anosmic (OR, 5.85 [95%CI 3.76, 9.10]) and hyposmic older adults (OR, 2.20 [95%CI 1.61, 3.02]) compared to those with normal olfactory function (Model A, Table 2).

**Table 1:** Demographic, olfactory and health characteristics of the population (N = 2,918).

	N	Estimated% of US Population
Mortality status at Wave 2 (% dead)	417	12.5
Odor identification (# of errors) a		
0	1,281	48.5
1	863	29.2
2	475	13.9
3	178	4.9
4	85	2.4
5	36	1.1
Sex (% men)	1,423	48.9
Race/ethnicity		
White	2,072	80.9
African American (AA)	487	9.8
Hispanic (non-AA)	291	6.8
Other	68	2.5
Education		
<High school (HS)	674	18.3
HS graduate or equiv.	765	26.8
Some college	832	30.1
Bachelors or higher	647	24.8
Disease Conditions		
Hypertension	1,676	54.0
Diabetes	630	19.9
Cancer^b^	365	12.6
Heart attack	349	11.6
Emphysema or COPD^c^	311	11.1
Heart failure	276	8.2
Stroke	261	8.1
Liver damage	33	1.1
Age (mean±SD^d^, range)		68.0±7.7, 57–85
Comorbidity Index (mean±SD^d^, range)		1.8±1.7, 0–10.5

a 0–1 error  =  Normosmic, 2–3 errors  =  Hyposmic, 4–5 errors  =  Anosmic.

b Excluding skin cancer.

c COPD  =  Chronic obstructive pulmonary disease.

d SD  =  standard deviation.

**Table 2:** Effects of olfactory dysfunction on death (logistic regression Model A), controlling for demographic variables (Model B), comorbidity index (Model C) and common diseases causing death (Model D) in older adults.

	*Odds Ratio, 95% Confidence Interval, p value*			
Covariates	Model A	Model B	Model C	Model D
Olfactory dysfunction (vs. Normosmic)^a^				
Anosmic	5.85^1^	3.24	3.41	3.37
	(3.76,9.10)^2^	(1.99,5.28)	(2.06,5.64)	(2.04,5.57)
	<0.001^3^	<0.001	<0.001	<0.001
				
Hyposmic	2.20	1.54	1.48	1.47
	(1.61,3.02)	(1.10,2.16)	(1.03,2.14)	(1.00,2.17)
	<0.001	0.01	0.04	0.05
				
				
Age (per year)		1.07	1.06	1.07
		(1.05,1.09)	(1.04,1.09)	(1.05,1.09)
		<0.001	<0.001	<0.001
				
				
Female gender		0.70	0.74	0.83
		(0.53,0.93)	(0.55,1.00)	(0.62,1.10)
		0.02	0.05	0.19
				
				
Race/ethnicity (vs. White)				
African American		0.91	0.88	0.86
		(0.65,1.27)	(0.62,1.24)	(0.60,1.23)
		0.58	0.45	0.40
				
Hispanic		0.57	0.61	0.58
		(0.36,0.89)	(0.36,1.02)	(0.34,0.99)
		0.01	0.06	0.05
				
Other		0.84	0.88	0.89
		(0.39,1.81)	(0.38,2.06)	(0.37,2.15)
		0.65	0.77	0.80
				
				
Education^b^		0.72	0.76	0.77
		(0.63,0.82)	(0.66,0.87)	(0.67,0.88)
		<0.001	<0.001	<0.001
				
				
Comorbidity Index			1.36	
			(1.28,1.45)	
			<0.001	
				
				
Heart attack				1.51
				(0.97,2.33)
				0.06
				
Heart failure				2.16
				(1.29,3.62)
				0.004
				
Stroke				2.04
				(1.51,2.76)
				<0.001
				
Diabetes				2.07
				(1.52,2.82)
				<0.001
				
Hypertension				0.84
				(0.64,1.10)
				0.20
				
Emphysema/COPD^c^				1.32
				(0.94,1.86)
				0.11
				
Liver damage				5.15
				(1.75,15.12)
				0.004
				
Cancer^d^				1.39
				(0.95,2.02)
				0.09

(N = 2,918; ^1^Odds ratio, ^2^95% Confidence interval, ^3^p value).

a 0–1 error  =  Normosmic, 2–3 errors  =  Hyposmic, 4–5 errors  =  Anosmic.

b Treated as a continuous measure using integer scores for educational level (higher scores  =  more education.).

c COPD  =  Chronic obstructive pulmonary disease.

d Excluding skin cancer.

Because these effects may simply reflect an association between olfaction and other factors increasing mortality, we then controlled for age (older people face higher odds of death), gender (men are more likely to die than women), socioeconomic status as measured by education level or assets (more educated or wealthier people are less likely to die), and race (racial health disparities may affect odds of death) (Model B, Table 2).

Older age increased the likelihood of death (OR, 1.07 [95%CI 1.05, 1.09]), whereas women had a lower likelihood of death (OR, 0.70 [95%CI 0.53, 0.93]) as did those with higher education (OR, 0.72 [95%CI 0.63, 0.82]); using assets yielded similar results. Despite adjusting for these demographic variables, those with anosmia still showed a substantially increased odds of death compared to those with normal olfactory function (OR, 3.24 [95%CI 1.99, 5.28]), and a smaller though statistically significant difference was also observed among hyposmic adults (OR, 1.54 [95%CI 1.10, 2.16]).

**Figure 2:**
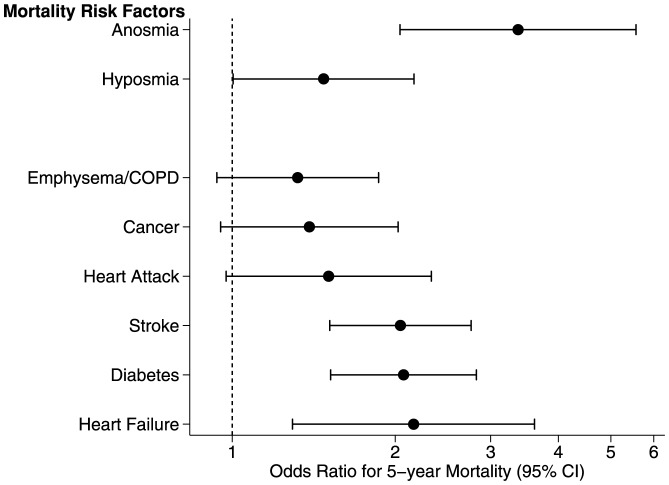
Odds for 5-year mortality for olfactory dysfunction compared to most common causes of death. Odds ratios with 95% confidence intervals are displayed in forest plot format. Confidence intervals that do not cross the vertical dashed line (odds ratio  = 1) indicate statistical significance at the 0.05 level. Heart attack refers to myocardial infarction, whereas heart failure refers to congestive heart failure.

Despite the possibility that medical conditions might account for these findings [22], the addition of a comorbidity index, a validated summary measure, did not reduce the magnitude of the effect (Model C, Table 2). Including specific diseases that are major risk factors for death in older adults (cardiovascular diseases, cancer, lung disease, stroke, diabetes and liver damage) (Model D, Table 2) did not attenuate the effect of olfactory function on mortality, confirming that our results were not an artifact of using a summary measure of comorbidity. Anosmia was a markedly stronger risk factor than most chronic diseases (See Figure 2).

The effect was strikingly robust, with anosmic adults retaining a high likelihood of dying in 5 years that was essentially unchanged (Model D: OR, 3.37 [95%CI 2.04, 5.57]). Increased comorbidity was associated with death (OR, 1.36 [95%CI 1.28, 1.45]) and individual conditions associated with death included heart failure (OR, 2.16 [95%CI 1.29, 3.62]), stroke (OR, 2.04 [95%CI 1.51, 2.76]), diabetes (OR, 2.07 [95%CI 1.52, 2.82]), and liver damage (OR, 5.15 [95%CI 1.75, 15.12]). Only severe liver damage was a stronger predictor of death than anosmia.

**Figure 3:**
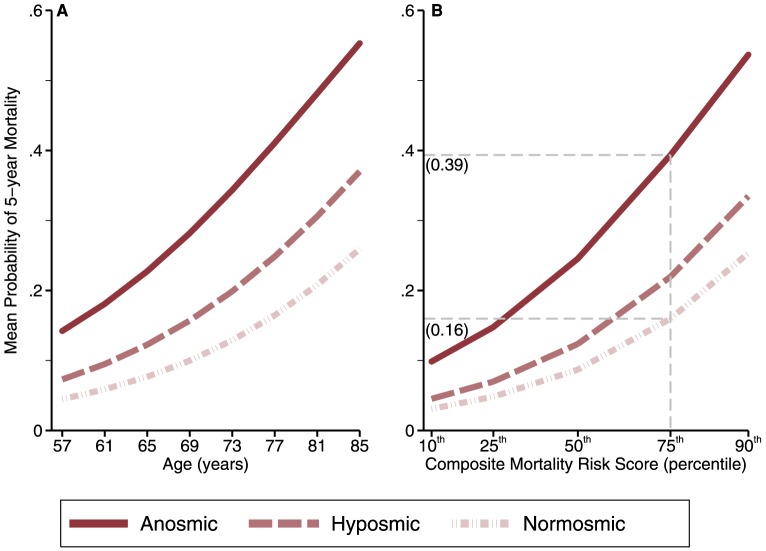
Effect of olfactory ability on the mean predicted probability of 5-year mortality, adjusting for age (A) and composite mortality risk score composed of all variables in Model C except olfaction (B). At the 75^th^ percentile of composite mortality risk, anosmia increases the average probability of death to 0.39 from 0.16 for normal smellers.

This effect was substantial enough to identify those at higher risk of mortality above and beyond other causes of death. Figure 3 shows the effect of anosmia beyond the effects of age alone (A) and age together with the other variables in Model C (B) on the probability of 5-year mortality, averaged across the entire population. For example, among those already at an elevated risk of death (at the 75^th^ percentile of composite risk score), anosmia increased the average probability of death to 0.39 from 0.16 for normal smellers.

To determine if the effect on mortality was being driven only by those with severe olfactory loss, we refit Model C using the number of olfactory errors to determine whether each additional error increased mortality risk (p<0.001 trend test, Figure 1B). Even when anosmics were excluded from the analysis, the number of errors was still associated with 5-year mortality (p = 0.041 trend test). Thus, even mild olfactory dysfunction was associated with increased odds of death. We also verified that mortality risk was not being driven by failure to detect one particular odor (Table S1 in File S1). Thus, lack of familiarity or culture bias did not explain our results.

### Potential Mechanisms

To test possible mechanisms, we added the corresponding variables to Model C to see if they accounted for the effects of olfactory dysfunction on mortality. First, we tested reduced nutrition by including self-reported taste function (recall that flavor perception is actually due to olfactory function), whether the respondent enjoyed eating, and BMI (Table S2 in File S1). Those who did not enjoy eating (OR, 1.22 [95%CI 1.01, 1.47]) or were underweight (OR, 3.88 [95%CI 1.29, 11.69]) were more likely to die after 5 years. However, those who were overweight had lower mortality (OR, 0.59 [95%CI 0.38, 0.92]), consistent with prior work [38]–[40], Nonetheless, including these variables in the model did not change the magnitude of the association between anosmia and mortality, which remained highly significant (OR, 3.29 [95%CI 1.78, 6.07]).

Olfaction and neurodegenerative diseases are linked clinically and pathologically, so we next determined whether cognitive function mediated the observed effect of an inability to identify odors on mortality. Those with impaired cognition were more likely to die (OR, 1.30 [95%CI 1.17, 1.44] per additional error on the SPMSQ). Controlling for cognitive function resulted in a small but significant diminution of the effect of anosmia on mortality (OR, 2.80 [95%CI 1.61, 4.86]) (Table S2 in File S1). This indicates cognitive deficits may be one small component explaining the effect of odor identification on mortality, but does not account for the majority of the role olfaction plays on this outcome.

Because mental health, and health behaviors, such as smoking and alcohol use, could also affect both olfaction and mortality, we included these in our models, with no reduction in the effect of anosmia on mortality (including mental health: OR, 3.43 [95%CI 2.06, 5.69]; including smoking and alcohol use: OR, 3.56 [95%CI 2.10, 6.05]) (Table S2 in File S1). We note that smoking causes increased mortality, and loss of olfactory function [42], although studies have not always found this effect on olfaction [41].

We then examined whether frailty, a predictor of mortality, mediated the relationship. Those who could not independently perform at least one activity of daily living had a higher risk of death compared to those without any ADL disability (OR, 2.22 [95%CI 1.63, 3.03]) (Table S2 in File S1), but controlling for ADL disability did not reduce the main effect of anosmia on death (OR, 3.69 [95%CI 2.22, 6.13]).

## Discussion

We are the first to show that olfactory dysfunction is a strong predictor of 5-year mortality in a nationally representative sample of older adults. Olfactory dysfunction was an independent risk factor for death, stronger than several common causes of death, such as heart failure, lung disease and cancer, indicating that this evolutionarily ancient special sense may signal a key mechanism that affects human longevity. This effect is large enough to identify those at a higher risk of death even after taking account of other factors, yielding a 2.4 fold increase in the average probability of death among those already at high risk (Figure 3B). Even among those near the median risk, anosmia increases the average probability of death from 0.09 (for normal smellers) to 0.25. Thus, from a clinical point of view, assessment of olfactory function would enhance existing tools and strategies to identify those patients at high risk of mortality.

We excluded several possibilities that might have explained these striking results. Adjusting for nutrition had little impact on the relationship between olfactory dysfunction and death. Similarly, accounting for cognition and neurodegenerative disease and frailty also failed to mediate the observed effects. Mental health, smoking, and alcohol abuse also did not explain our findings. Risk factors for olfactory loss (male gender, lower socioeconomic status, BMI) were included in our analyses, and though they replicated prior work [41], did not affect our results.

Our study does have a few notable limitations. Because our 5-item olfactory test is less reliable than a longer assessment, our results likely represent an underestimate of the magnitude of the association between olfactory dysfunction and mortality. In addition, the home setting in which the interviews were conducted precluded assessments of physiological and anatomical characteristics typically performed in the clinic or hospital. Finally, our dataset does not include cause of death, data that would permit additional exploration of specific mechanisms.

We believe olfaction is the canary in the coalmine of human health, not that its decline directly causes death. Olfactory dysfunction is a harbinger of either fundamental mechanisms of aging, environmental exposure, or interactions between the two. Unique among the senses, the olfactory system depends on stem cell turnover, and thus may serve as an indicator of deterioration in age-related regenerative capacity more broadly or as a marker of physiologic repair function [13].

The olfactory nerve is the only cranial nerve directly exposed to the environment. Thus, it is possible that respiratory exposures (pollution, toxins, pathogens) could reach the central nervous system via the olfactory nerve and cause death due to direct injurious effects, consistent with the olfactory vector hypothesis of neurodegenertives diseases[43], [44]. Alternatively, these exposures could be absorbed and cause systemic effects (e.g., pulmonary, cardiovascular systems) resulting in death as they simultaneously injure the olfactory epithelium. Particulate matter pollution has been shown to increase morbidity and mortality and decrease olfaction, although the precise mechanism of these effects is unknown [45]. Further investigation is required to distinguish which of these etiologies may explain our results. Another open question for future work is whether this relationship is present in younger adults.

The test we employed here is a shortened version of a standard olfactory test used clinically, taking only ∼3 minutes to deploy and score. Despite this, our method is able to distinguish groups with substantial differences in mortality. Thus, this short olfactory test may have clinical utility in identifying patients at risk and who might benefit from additional clinical scrutiny and further follow-up. Our results extend the existing literature on biomarkers of mortality including functional and physiological measures, with the ability of olfaction to predict death as large as, if not exceeding, other biomarkers [46]–[53]. To our knowledge, no previous study has examined sensory function as a predictor of mortality in a nationally representative population sample. Thus, our results have broad implications for understanding mortality of older adults in the US and worldwide.

## Supporting Information

File S1 **This file contains Table S1 and Table S2.** Table S1, Logistic regressions, each excluding one odor to determine if it in particular was driving the effect (adjusting for age, gender, race/ethnicity, education and comorbidity index; N = 2,918). Table S2, Factors possibly mediating the effect of olfactory dysfunction on mortality: nutrition, cognition, mental health, health behaviors, and frailty.
(DOCX)
