# Supplementary material for: Olfactory Dysfunction Predicts 5-Year Mortality in Older Adults
Source: PLoS One. 2014 Oct 1;9(10):e107541. doi: 10.1371/journal.pone.0107541 (PMC4182669; doi:10.1371/journal.pone.0107541)
Supplement: File S1 — This file contains Table S1 and Table S2. Table S1, Logistic regressions, each excluding one odor to determine if it in particular was driving the effect (adjusting for age, gender, race/ethnicity, education and comorbidity index; N = 2,918). Table S2, Factors possibly mediating the effect of olfactory dysfunction on mortality: nutrition, cognition, mental health, health behaviors, and frailty. (DOCX) [file pone.0107541.s001.docx]

Table S1. Logistic regressions, each excluding one odor to determine if it in particular was driving the effect (adjusting for age, gender, race/ethnicity, education and comorbidity index; N=2,918)

Table S2. Factors possibly mediating the effect of olfactory dysfunction on mortality: nutrition, cognition, mental health, health behaviors, and frailty

**Table S1. Logistic regressions, each excluding one odor to determine if it in particular was driving the effect (adjusting for age, gender, race/ethnicity, education and comorbidity index; N=2,918). ^1^Odds ratio, ^2^95% Confidence Interval.**

|  | **Odds Ratio and 95% Confidence Interval** | | | | |
| --- | --- | --- | --- | --- | --- |
|  |  |  |  |  |  |
|  | **Excluding Rose** | **Excluding**  **Leather** | **Excluding**  **Orange** | **Excluding**  **Fish** | **Excluding**  **Peppermint** |
| Odor identification  (# of errors, vs. 0) |  |  |  |  |  |
| 1 | 1.15^1^  (0.84, 1.57) ^2^ | 1.39  (0.97, 1.99) | 1.10  (0.78, 1.54) | 1.07  (0.79, 1.45) | 1.23  (0.90, 1.68) |
| 2 | 1.46  (0.96, 2.20) | 1.69  (1.10, 2.62) | 1.53  (0.99, 2.37) | 1.59  (1.02, 2.49) | 1.42  (0.91, 2.21) |
| 3 | 2.51  (1.34, 4.69) | 2.55  (1.55, 4.18) | 2.28  (1.25, 4.18) | 2.25  (1.33, 3.82) | 2.11  (1.32, 3.38) |
| 4 | 3.95  (1.99, 7.84) | 5.10  (2.37, 11.00) | 3.52  (1.69, 7.33) | 3.26  (1.66, 6.39) | 5.71  (2.60, 12.54) |
|  |  |  |  |  |  |
| Overall p value ^a^ | <0.001 | <0.001 | <0.001 | <0.001 | <0.001 |

^a^ One degree of freedom test for trend.

**Table S2. Factors possibly mediating the effect of olfactory dysfunction on mortality: nutrition, cognition, mental health, health behaviors, and frailty. ^1^Odds ratio (95% Confidence Interval), ^2^p value.**

|  | **Odds Ratio, 95% Confidence Interval, p value** | | | | | |
| --- | --- | --- | --- | --- | --- | --- |
|  |  |  |  |  |  |  |
| **Covariates** | **Model C** | **Model C + Nutrition** | **Model C + Cognition** | **Model C +**  **Mental Health** | **Model C +**  **Health Behaviors** | **Model C +**  **Frailty** |
| Olfactory dysfunction  (vs. Normosmic) |  |  |  |  |  |  |
| Anosmic | 3.41 (2.06,5.64) ^1^ | 3.29 (1.78,6.07) | 2.80 (1.61,4.86) | 3.43 (2.06,5.69) | 3.56 (2.10,6.05) | 3.69 (2.22,6.13) |
|  | <0.001^2^ | <0.001 | <0.001 | <0.001 | <0.001 | <0.001 |
|  |  |  |  |  |  |  |
| Hyposmic | 1.48 (1.03,2.14) | 1.56 (0.88,2.78) | 1.36 (0.95,1.96) | 1.45 (1.00,2.10) | 1.48 (1.03,2.13) | 1.40 (0.97,2.01) |
|  | 0.04 | 0.13 | 0.09 | 0.05 | 0.03 | 0.07 |
|  |  |  |  |  |  |  |
|  |  |  |  |  |  |  |
| Age (per year) | 1.06 (1.04,1.09) | 1.05 (1.02,1.08) | 1.06 (1.04,1.08) | 1.06 (1.04,1.08) | 1.08 (1.06,1.10) | 1.06 (1.04,1.08) |
|  | <0.001 | 0.001 | <0.001 | <0.001 | <0.001 | <0.001 |
|  |  |  |  |  |  |  |
|  |  |  |  |  |  |  |
| Female gender | 0.74 (0.55,1.00) | 0.70 (0.49,1.01) | 0.71 (0.52,0.96) | 0.72 (0.54,0.97) | 0.80 (0.59,1.09) | 0.67 (0.50,0.90) |
|  | 0.05 | 0.06 | 0.03 | 0.03 | 0.15 | 0.009 |
|  |  |  |  |  |  |  |
|  |  |  |  |  |  |  |
| Race/ethnicity  (vs. White) |  |  |  |  |  |  |
| African American | 0.88 (0.62,1.24) | 0.78 (0.45,1.35) | 0.77 (0.55,1.09) | 0.85 (0.59,1.21) | 0.85 (0.60,1.20) | 0.85 (0.60,1.20) |
|  | 0.45 | 0.36 | 0.14 | 0.36 | 0.36 | 0.35 |
|  |  |  |  |  |  |  |
| Hispanic | 0.61 (0.36,1.02) | 0.37 (0.18,0.75) | 0.49 (0.27,0.90) | 0.54 (0.32,0.91) | 0.68 (0.40,1.17) | 0.58 (0.33,1.03) |
|  | 0.06 | 0.007 | 0.02 | 0.02 | 0.16 | 0.06 |
|  |  |  |  |  |  |  |
| Other | 0.88 (0.38,2.06) | 0.92 (0.33,2.56) | 0.80 (0.34,1.85) | 0.85 (0.40,1.83) | 0.82 (0.35,1.89) | 0.86 (0.37,2.02) |
|  | 0.77 | 0.87 | 0.59 | 0.68 | 0.63 | 0.73 |
|  |  |  |  |  |  |  |
|  |  |  |  |  |  |  |
| Education^a^ | 0.76 (0.66,0.87) | 0.74 (0.62,0.88) | 0.80 (0.69,0.92) | 0.78 (0.68,0.90) | 0.79 (0.69,0.90) | 0.79 (0.68,0.90) |
|  | <0.001 | 0.001 | 0.003 | 0.001 | 0.001 | 0.001 |
|  |  |  |  |  |  |  |
|  |  |  |  |  |  |  |
|  | **Model C** | **Model C + Nutrition** | **Model C + Cognition** | **Model C +**  **Mental Health** | **Model C +**  **Health Behaviors** | **Model C +**  **Frailty** |
|  |  |  |  |  |  |  |
| Comorbidity Index | 1.36 (1.28,1.45) | 1.40 (1.27,1.54) | 1.36 (1.27,1.45) | 1.34 (1.26,1.43) | 1.36 (1.28,1.46) | 1.29 (1.20,1.38) |
|  | <0.001 | <0.001 | <0.001 | <0.001 | <0.001 | <0.001 |
|  |  |  |  |  |  |  |
|  |  |  |  |  |  |  |
| BMI  (vs. Normal weight) |  |  |  |  |  |  |
| Underweight |  | 3.88 (1.29,11.69) |  |  |  |  |
|  |  | 0.02 |  |  |  |  |
|  |  |  |  |  |  |  |
| Overweight/obese |  | 0.59 (0.38,0.92) |  |  |  |  |
|  |  | 0.02 |  |  |  |  |
|  |  |  |  |  |  |  |
| Unknown |  | 2.12 (1.11, 4.04) |  |  |  |  |
|  |  | 0.02 |  |  |  |  |
|  |  |  |  |  |  |  |
| Self-reported taste^b^ |  | 1.15 (0.93,1.41) |  |  |  |  |
|  |  | 0.18 |  |  |  |  |
|  |  |  |  |  |  |  |
| Poor appetite^c^ |  | 1.22 (1.01,1.47) |  |  |  |  |
|  |  | 0.04 |  |  |  |  |
|  |  |  |  |  |  |  |
|  |  |  |  |  |  |  |
| SPMSQ^d^  (# of errors) |  |  | 1.30 (1.17,1.44) |  |  |  |
|  |  |  | <0.001 |  |  |  |
|  |  |  |  |  |  |  |
|  |  |  |  |  |  |  |
| Self-reported mental health^e^ |  |  |  | 1.20 (1.06,1.36) |  |  |
|  |  |  |  | 0.006 |  |  |
|  |  |  |  |  |  |  |
|  |  |  |  |  |  |  |
| Current smoker |  |  |  |  | 2.39 (1.73,3.31) |  |
|  |  |  |  |  | <0.001 |  |
|  |  |  |  |  |  |  |
| Problem drinking |  |  |  |  | 1.14 (0.83,1.57) |  |
|  |  |  |  |  | 0.40 |  |
|  | **Model C** | **Model C + Nutrition** | **Model C + Cognition** | **Model C +**  **Mental Health** | **Model C +**  **Health Behaviors** | **Model C +**  **Frailty** |
|  |  |  |  |  |  |  |
| ADL^f^ disability |  |  |  |  |  | 2.22 (1.63,3.03) |
|  |  |  |  |  |  | <0.001 |
|  |  |  |  |  |  |  |
| N | 2,918 | 1,455 | 2,918 | 2,909 | 2,918 | 2,912 |

^a^ Treated as a continuous measure using integer scores for educational level (higher scores = more education)

^b^ Treated as a continuous measure using integer scores for taste level (higher scores = worse taste)

^c^ Based on response to “During the past week…I did not feel like eating; my appetite was poor.” Response choices: rarely/none of the time, some of the time, occasionally, most of the time. Treated as a continuous measure using integer scores for frequency (higher scores = greater frequency)

^d^ SPMSQ = Short Portable Mental Status Questionnaire

^e^ Treated as a continuous measure using integer scores for mental health level (higher scores = worse health)

^f^ ADL = Activities of Daily Living
